# Prevalence of hypertension in endemic and non-endemic areas of Keshan disease: A cross-sectional study in rural areas of China

**DOI:** 10.3389/fnut.2023.1086507

**Published:** 2023-02-13

**Authors:** Jie Hou, Lifang Zhu, Shuran Jin, Jinshu Li, Zhifeng Xing, Yanling Wang, Xiaoyan Wan, Xianni Guo, Anwei Wang, Xiuhong Wang, Jinming Liu, Jing Ma, Shuang Zhou, Xiangdong Zhang, Heming Zheng, Jianhui Wang, Hongqi Feng, Shuqiu Sun, Tong Wang

**Affiliations:** ^1^Institute of Keshan Disease, Center for Endemic Disease Control, Chinese Center for Disease Control and Prevention, Harbin Medical University, Harbin, China; ^2^National Health Commission and Education Bureau of Heilongjiang Province, Key Laboratory of Etiology and Epidemiology, Harbin Medical University, Harbin, China; ^3^Sichuan Center for Disease Control and Prevention, Chengdu, China; ^4^Heilongjiang Provincial Center for Disease Control and Prevention, Harbin, China; ^5^Gansu Provincial Center for Disease Control and Prevention, Lanzhou, China; ^6^The Second Research Institute for Endemic Disease Control and Prevention of Jilin Province, Jilin City, China; ^7^Shaanxi Institute for Endemic Disease Control and Prevention, Xi’an, China; ^8^Yunnan Institute of Endemic Disease Control and Prevention, Dali, China; ^9^Shandong Provincial Institute for Endemic Disease Control, Jinan, China; ^10^The Inner Mongolia Autonomous Region Comprehensive Center for Disease Control and Prevention, Hohhot, China; ^11^Hebei Provincial Center for Disease Control and Prevention, Shijiazhuang, China; ^12^Chongqing Center for Disease Control and Prevention, Chongqing, China; ^13^Shanxi Institute of Endemic Disease Control and Prevention, Linfen, China; ^14^Henan Provincial Center for Disease Control and Prevention, Zhengzhou, China; ^15^Liaoning Center for Disease Control and Prevention, Shenyang, China

**Keywords:** hypertension, Keshan disease, cross-sectional study, blood pressure, selenium, diet

## Abstract

**Background:**

Hypertension is a major public health concern that strongly influences the quality of life of people worldwide. Keshan disease (KD) is an endemic cardiomyopathy related to low selenium, threatening residents in rural areas of 16 provinces in China. Furthermore, the prevalence of hypertension in the KD-endemic areas has been increasing annually. However, hypertension research associated with KD has only focused on endemic regions, and no studies have compared hypertension prevalence between endemic and non-endemic areas. Therefore, this study investigated the prevalence of hypertension to provide a basis for preventing and controlling hypertension in the KD-endemic areas, even in rural areas.

**Methods:**

We extracted blood pressure information from cardiomyopathy investigation data from a cross-sectional study of the KD-endemic and non-endemic areas. The hypertension prevalence between the two groups was compared using the Chi-square test or Fisher s exact test. Additionally, Pearson’s correlation coefficient was employed to evaluate the relationship between the per capita gross domestic product (GDP) and hypertension prevalence.

**Results:**

There was a statistically significant increase of hypertension prevalence in the KD-endemic areas (22.79%, 95% confidence interval [CI]: 22.30–23.27%) over the non-endemic areas (21.55%, 95% CI: 21.09–22.02%). In the KD-endemic areas, more men had hypertension than women (23.90% vs. 21.65%, *P* < 0.001). Furthermore, the hypertension prevalence was higher in the north than in the south in the KD-endemic areas (27.52% vs. 18.76%, *P* < 0.001), non-endemic areas (24.86% vs. 18.66%, *P* < 0.001), and overall (26.17% vs. 18.68%, *P* < 0.001). Finally, the prevalence of hypertension positively correlated with per capita GDP at province level.

**Conclusions:**

The increasing hypertension prevalence is a public health problem in the KD-endemic areas. Healthy diets, such as high consumption of vegetables and seafoods, and foods that are rich in selenium, might help prevent and control hypertension in the KD-endemic areas and other rural areas in China.

## 1. Introduction

More than 200 million adults have hypertension in China, accounting for over one-fifth of China’s adult population (1). Moreover, hypertension is a major public health concern worldwide and is a risk factor for abundant diseases, such as cardiovascular (2, 3) and kidney diseases (4). More than half of the global adult population have not been diagnosed with or treated for hypertension. Consequently, only approximately 20% of adult patients have experienced hypertension control through medical care (4). Moreover, residents of rural areas may be at higher risk than those in urban areas in low-and middle-income countries.

Keshan disease (KD) is an endemic cardiomyopathy characterized by degeneration, necrosis, and fibrosis of cardiomyocytes, and heart dilatation, threatening the residents of rural areas in 16 provinces of China (5). In addition, hair and serum samples from individuals from these populations and soil and grain samples from the KD-endemic areas indicated low selenium levels (6, 7). Moreover, Mihailović et al. (8) found that patients with arterial hypertension had significantly lower whole-blood and plasma selenium concentrations.

A 10-year follow-up study verified that hypertension is a risk factor for latent KD worsening into chronic KD (9). Recently, with economic development and changes in diet, the prevalence of hypertension in the KD-endemic areas has increased annually (10), exceeding the national average (11). Previous hypertension research associated with KD has focused on the endemic areas, whilst no studies have investigated hypertension disparities between the KD-endemic and non-endemic areas. Therefore, this study used the blood pressure data from a cardiomyopathy investigation of residents of the KD-endemic and non-endemic areas in 2011 to understand the prevalence of hypertension and provide a base for preventing and controlling hypertension in the KD-endemic areas, even in the rural areas in China.

## 2. Materials and methods

### 2.1. Multistage cluster sampling

We extracted data from a cross-sectional study comprising KD surveillance in KD-endemic counties and dilated cardiomyopathy surveyed in non-endemic counties in 13 provinces. The provinces included Heilongjiang, Nei Mongol, Jilin, Gansu, Shaanxi, Liaoning, Shanxi, Shandong, Henan, Hebei, Yunnan, Sichuan, and Chongqing. There are more endemic counties and higher KD prevalence in the 13 provinces among 16 provinces affected by KD in China. KD surveillance has been gradually conducted in those provinces since 1990. The other three provinces, Hubei, Guizhou and Tibet, were excluded due to only one KD county and few KD cases occurred. The KD-endemic areas were determined using the Delimitation and Classification of Keshan Disease Areas (GB17020-2010) (12). In this study, we used multistage cluster sampling. For each county, we performed the case search (6) to identify two townships with the most patients with KD or dilated cardiomyopathy. Then, we selected the village with the most patients in either of the two townships for the investigation. The included villages had populations greater than or equal to 500 people. The endemic and non-endemic counties were individually matched based on the geographical location and residents’ lifestyles. Finally, hypertension data were collected from the 49 KD-endemic and 49 non-endemic counties.

### 2.2. Participants

All village residents underwent medical examinations, including blood pressure and electrocardiograms. After, patients with suspected KD or dilated cardiomyopathy were examined using echocardiography and chest radiography. We required a response rate of 80% or higher or at least 400 surveyed individuals. If the quantity did not meet these requirements, it would be supplemented by the neighboring village. All included participants had lived in the surveyed village for more than 6 consecutive months or had left for no more than 3 months in the past year. We examined 43,240 and 104,166 people in the KD-endemic and non-endemic areas, respectively. Then, we extracted blood pressure data for 58,994 participants aged 20 years or older for the analysis.

### 2.3. Blood pressure measurements

After sitting in a relaxed position comfortably and quietly for more than 5 min, blood pressure was measured using a mercury sphygmomanometer. The participants were informed that smoking, drinking, and other activities resulting in blood pressure instability were forbidden for at least 30 min before the measurement. During the measurement, the elbow and forearm were bent flush with the heart, and the cuff was placed on the right bare upper arm one inch above the bend of the elbow with appropriate tightness. The disk of the stethoscope was placed face down under the cuff, just to the inner side of the upper arm, where the brachial artery pulse could be felt. The cuff was rapidly inflated until the pulse voice disappeared and continued to be pressurized until it was slowly deflated after the gauge reading had risen by 20–30 mmHg. The first loud beat heard was the systolic blood pressure (SBP), and the last beat before it disappeared was the diastolic blood pressure (DBP).

### 2.4. Economic and demographic data

Demographic data including age and sex and the per capita gross domestic product (GDP) were collected for each province in 2011 from the 2012 China Statistical Yearbook (13).

### 2.5. Ethics

All participants signed an informed statement to give permission and indicate that they had no direct interest in the study’s results. This study conformed to the Declaration of Helsinki and has been authorized by the Medical Ethics Committee of Harbin Medical University.

### 2.6. Statistical analyses

The statistical analyses were executed with R Studio version 1.4.1717.^1^ Hypertension was defined as either a SBP of 140 mmHg or greater or a DBP of 90 mmHg or greater or the presence of both based on the 2019 Annual Report on Cardiovascular Health and Diseases in China (14). Hypertension was classified into three categories: Grade 1 (140–159/90–99 mmHg), Grade 2 (160–179/100–109 mmHg), and Grade 3 (≥180/110 mmHg) (15). We excluded SBP, DBP, or pulse pressure data outside a 99.73% confidence interval (CI). We also screened for duplicated records and, if identified, randomly retained one of the duplicates. Repeat data were defined as consistent information, including province, county, township, age, sex, SBP, DBP, pinyin of name, and telephone number.

The distributions of the participant characteristics were described using the population pyramid. Bar charts were used to depict the prevalence of hypertension in the KD-endemic and non-endemic areas by age categories. Forest plots were employed to describe the hypertension prevalence rate at the province level, while error bars with 95% CIs were used to demonstrate hypertension differences between the sexes. The prevalence was standardized by age and sex based on the 2012 China Statistical Yearbook (13). The prevalence between the two groups was compared using the Chi-square test or Fisher’s exact test, and the relationship between the per capita GDP and hypertension prevalence at province level was analyzed using Pearson’s correlation coefficient. The statistical significance was delimited at *P* < 0.05.

## 3. Results

### 3.1. Total hypertension prevalence

**FIGURE 1:**
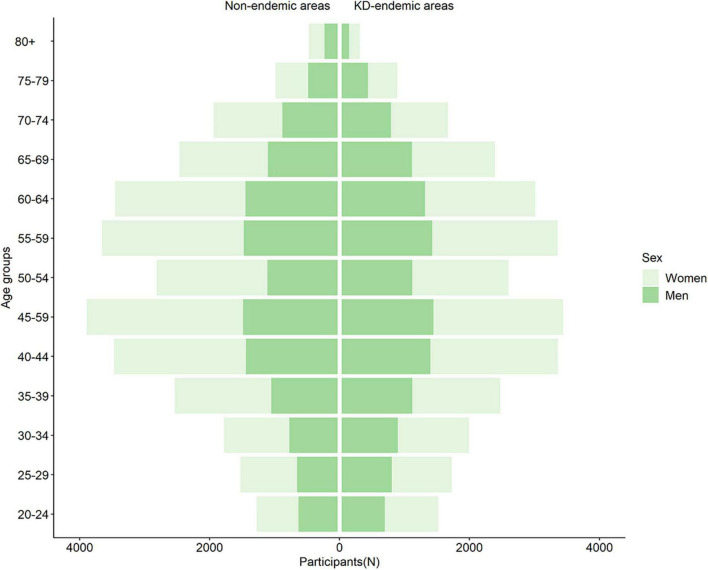
Population pyramid of participants in KD-endemic and non-endemic areas. KD, Keshan disease; N, number.

We recruited 58,994 participants, including 28,738 participants from the KD-endemic areas and 30,256 from the non-endemic areas. Figure 1 presents the age and sex distributions of the respondents. The prevalence of hypertension was higher in the KD-endemic areas (22.79%, 95% CI: 22.30–23.27%) than in the non-endemic areas (21.55%, 95% CI: 21.09–22.02%, *P* < 0.001, Table 1).

**TABLE 1:** Prevalence of hypertension in KD-endemic and non-endemic areas.

KD areas	Participants	Patients with hypertension	Prevalence (95% CI)	Age, sex-standardized prevalence (95% CI)
Endemic	28,738	8,172	28.44% (27.92–28.96%)	22.79% (22.30–23.27%)^a^
Non-endemic	30,256	8,364	27.64% (27.14–28.15%)	21.55% (21.09–22.02%)

CI, confidence interval; KD, Keshan disease.

^a^*P* < 0.001 compared with the non-endemic areas.

### 3.2. Hypertension prevalence by age categories, sex, and grade

**FIGURE 2:**
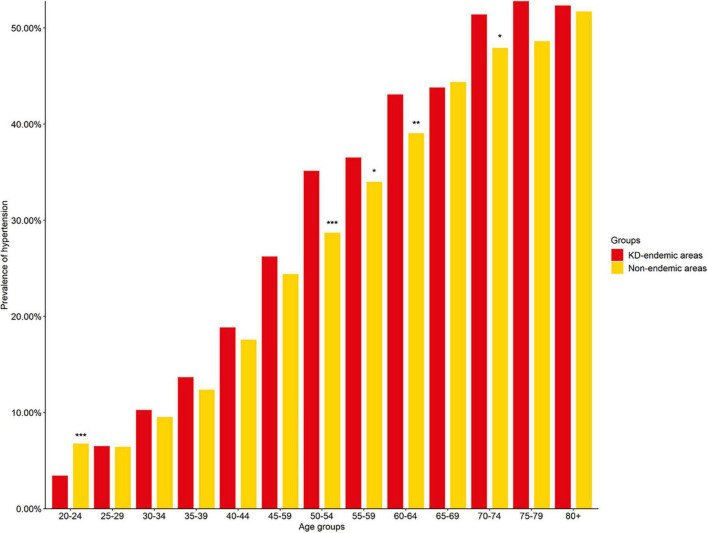
Hypertension prevalence in KD-endemic and non-endemic areas by age groups. The prevalence was standardized by age and sex based on the 2012 China Statistical Yearbook. KD. Keshan disease; *P < 0.05 compared with KD-endemic areas; **P < 0.01 compared with KD-endemic areas; ***P < 0.001 compared with KD-endemic areas.

Hypertension prevalence increased with age; the highest prevalence was in the 75–79 and 80+ year age groups in the endemic and non-endemic areas, respectively. Furthermore, in four age groups (50–54, 55–59, 60–64, and 70–74 years), the prevalence of hypertension was lower in the non-endemic areas than in the KD-endemic areas (*P* < 0.05, Figure 2 and Supplementary Table 1).

**FIGURE 3:**
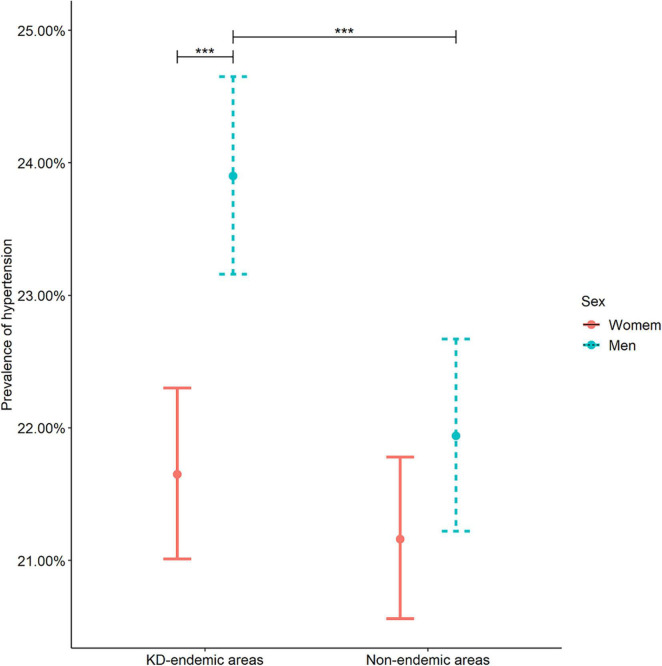
Hypertension prevalence in KD-endemic and non-endemic areas by sex. Error bars with 95% CIs were used to demonstrate hypertension differences between the sexes. The prevalence was standardized by age and sex based on the 2012 China Statistical Yearbook. KD, Keshan disease; ***P < 0.001 compared with women in the KD-endemic areas or men in the non-endemic areas.

The prevalence of hypertension among men in the KD-endemic areas exceeded that among men in the non-endemic areas and women in the KD-endemic areas (*P* < 0.001, Figure 3). The prevalence of Grade 1 and 2 hypertension was higher in the KD-endemic areas than in the non-endemic areas, whereas the prevalence of Grade 3 hypertension was the opposite (*P* < 0.001, Table 2).

**TABLE 2:** Prevalence of hypertension grades in KD-endemic and non-endemic areas.

Hypertension grade	KD areas	Participants	Patients with hypertension	Prevalence (95% CI)	Age, sex-standardized prevalence (95% CI)
Grade 1	Endemic	28,738	5,501	19.14% (18.69–19.60%)	15.79% (15.37–16.22%)^a^
	Non-endemic	30,256	5,455	18.03% (17.60–18.48%)	14.74% (14.34–15.15%)
Grade 2	Endemic	28,738	2,025	7.05% (6.75–7.35%)	5.36% (5.10–5.61%)^a^
	Non-endemic	30,256	2,011	6.65% (6.37–6.93%)	4.74% (4.50–4.99%)
Grade 3	Endemic	28,738	646	2.25% (2.08–2.46%)	1.65% (1.51–1.80%)^a^
	Non-endemic	30,256	898	2.97% (2.78–3.17%)	2.08% (1.92–2.25%)

Grade 1, 140–159/90–99 mmHg; Grade 2, 160–179/100–109 mmHg; Grade 3, ≥180/110 mmHg; CI, confidence interval; KD, Keshan disease.

^a^*P* < 0.001 compared with the non-endemic areas.

### 3.3. Hypertension prevalence by region

The annual average temperature of the capital cities in the provinces included in this investigation was 11.7°C. Therefore, provinces with an annual average temperature above 11.7°C were classified as being in the south and included Shandong, Shaanxi, Henan, Hebei, Yunnan, Sichuan, and Chongqing. Conversely, provinces with an annual average temperature below 11.7°C were classified as being in the north and included Heilongjiang, Jilin, Liaoning, Nei Mongol, Shanxi, and Gansu.

In the north, the prevalence of hypertension was significantly higher in the KD-endemic areas than in the non-endemic areas (*P* < 0.001). Moreover, the prevalence of hypertension was significantly higher in the north than in the south, regardless of the endemic classification (*P* < 0.001, Table 3).

**TABLE 3:** Prevalence of hypertension in KD-endemic and non-endemic areas in the southern and northern regions of China.

Region	KD areas	Participants	Patients with hypertension	Prevalence (95% CI)	Age, sex-standardized prevalence (95% CI)
South	Endemic	15,741	3,727	23.68% (23.01–24.35%)	18.76% (18.15–19.38%)
	Non-endemic	16,043	3,962	24.70% (24.03–25.37%)	18.66% (18.06–19.27%)
	Total	31,784	7,689	24.19% (23.72–24.67%)	18.68% (18.25–19.11%)
North	Endemic	12,997	4,445	34.20% (33.38–35.02%)	27.52% (26.76–28.30%)^a,b^
	Non-endemic	14,213	4,402	30.97% (30.21–31.74%)	24.86% (24.15–25.58%)^b^
	Total	27,210	8,847	32.51% (31.96–33.07%)	26.17% (25.65–26.70%)^b^

South, provinces with an annual average temperature higher than 11.7°C, including Shandong, Shaanxi, Henan, Hebei, Yunnan, Sichuan, and Chongqing; North, provinces with an annual average temperature lower than 11.7°C, including Heilongjiang, Jilin, Liaoning, Nei Mongol, Shanxi, and Gansu; CI, confidence interval; KD, Keshan disease.

^a^*P* < 0.001 compared with the non-endemic areas in the north.

^b^*P* < 0.001 compared with the south.

### 3.4. Hypertension prevalence by province

**FIGURE 4:**
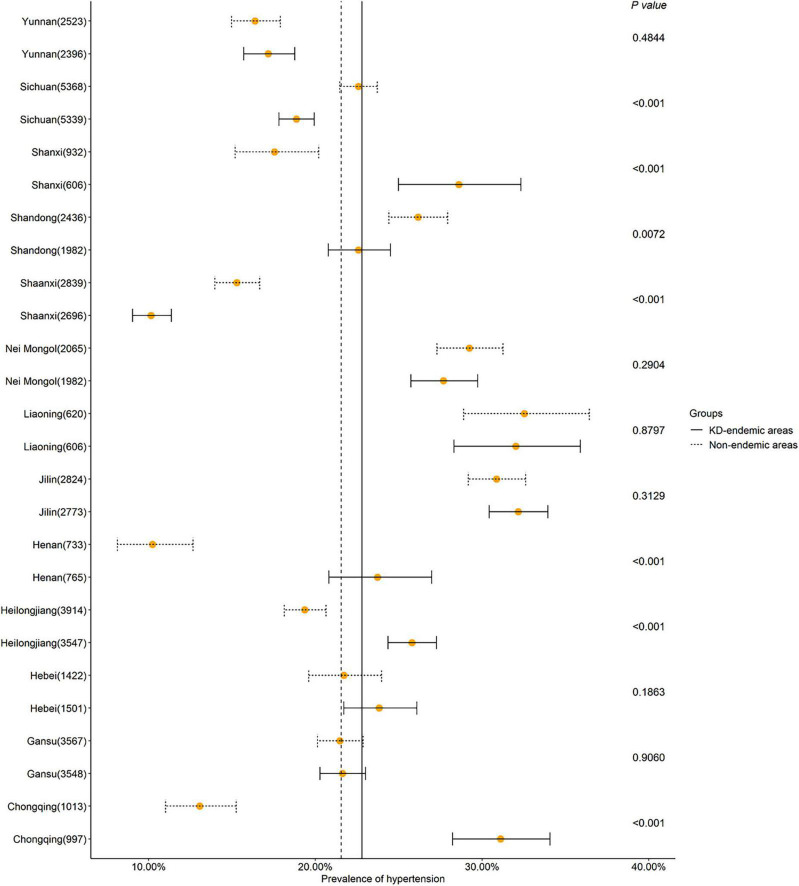
Hypertension prevalence in KD-endemic and non-endemic areas by province. Forest plots were employed to describe the hypertension prevalence rate at the province level. The prevalence was standardized by age and sex based on the 2012 China Statistical Yearbook. KD, Keshan disease; The number in the bracket represents the total number of participants in each province. The upright solid line indicates the hypertension prevalence in the KD-endemic areas in total. The upright dotted line indicates the hypertension prevalence in the non-endemic areas in total.

In the Shanxi, Henan, Heilongjiang, and Chongqing provinces, the prevalence of hypertension was significantly lower in the non-endemic areas than in the endemic areas (*P* < 0.001, Figure 4 and Supplementary Table 2). However, the opposite was observed in the Sichuan (*P* < 0.001), Shandong (*P* < 0.001), and Shaanxi (*P* < 0.05) provinces.

### 3.5. Hypertension prevalence and per capita GDP

**FIGURE 5:**
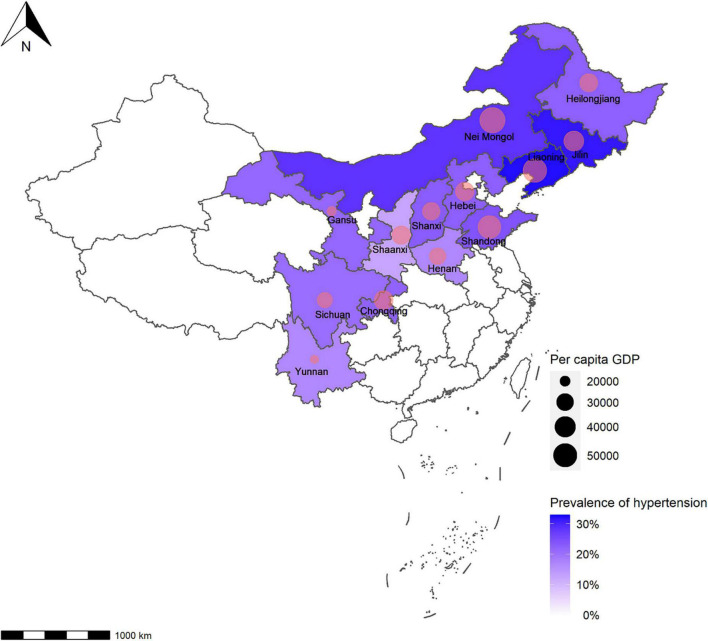
Hypertension prevalence and per capita GDP at province level. The relationship between the per capita GDP and hypertension prevalence at province level was analyzed using Pearson’s correlation coefficient (*r* = 0.6672, *P* = 0.0127). The prevalence was standardized by age and sex based on the 2012 China Statistical Yearbook. GDP, gross domestic product.

Pearson’s correlation coefficient was determined to be *r* = 0.6672 (*P* = 0.0127), suggesting that the prevalence of hypertension positively correlated with the per capita GDP by province (Figure 5, Supplementary Table 3 and Supplementary Figure 1).

## 4. Discussion

We conducted a large-scale and representative study, reporting for the first time that the prevalence of hypertension is higher in the KD-endemic areas than in the non-endemic areas. This may be related to the suboptimal selenium intake of residents in the KD-endemic areas. The Western European longitudinal population study demonstrated that a 20 μg/L or higher blood selenium level at baseline reduced the risk of hypertension by 37% in men (16), and adults with low toenail selenium concentrations had an increased risk of hypertension (17). Furthermore, Xie et al. (18) reported a negative correlation between selenium intake and hypertension in participants in northern provinces but a positive correlation in participants in southern provinces in China. Lower urinary selenium concentrations were also associated with higher SBP and DBP values in Asian countries (19), similar to the associations identified between the serum selenium level and SBP and DBP in pregnant women (20). However, some studies have suggested a positive correlation between selenium levels and hypertension (21–23), which may be due to the presence of high levels of selenium in the areas investigated. About half of the Chinese population does not meet the recommended selenium intake defined by the Food and Agriculture Organization and the World Health Organization (24). Therefore, increasing the intake of selenium-rich foods might be beneficial for the residents of low-selenium areas, reducing the prevalence of hypertension.

Men are more prone to hypertension than women (25–27), which is consistent with our study’s results (Supplementary Figures 2, 3). We found that the prevalence of hypertension was higher in men than in women in the KD-endemic areas. Everett and Zajacova (28) reported that among Americans aged 24–34 years, women were far less likely to be hypertensive because they were more aware of hypertension than men. Women had more advanced hypertension awareness than men in China (1), the United States (29), and Romania (30). Meanwhile, men consumed alcohol in larger amounts and more frequently than women (31). After stratifying by sex, daily drinking increased the risk of hypertension in men but did not affect women in Southwest China (32). A J-shaped relationship between alcohol consumption and hypertension has been identified in women, while alcohol consumption was linearly correlated with the risk of hypertension in men (33, 34). These studies indicate that alcohol consumption could be the reason for a higher hypertension prevalence in men.

We found the hypertension prevalence was higher in the north than in the south, perhaps highlighting the role of temperature. A previous study reported that the prevalence of hypertension and the average SBP and DBP in northern tourists in Hainan, located in one of the most southern regions of China, were significantly higher than those of local residents and northern residents living in Hainan for more than 5 years (35). Moreover, Duranton et al. (36) collected data from 261 hemodialysis patients in different latitudes, discovering that the rising outdoor temperatures and prolonged sunshine hours were associated with decreased blood pressure before dialysis. When the temperature dropped by 1°C, the SBP and DBP for the total population rose by 0.55 and 0.26 mmHg, respectively (37). Moreover, residents in the north of China had 2.32 g more sodium daily than those in the south of China (38). One study reported that individuals with hypertension or normal blood pressure could lower their blood pressure by moderately reducing their salt intake for 4 weeks or more (39). Another study reported a reduction in SBP and DBP by 1.10 mmHg and 0.33 mmHg, respectively, for every 50 mmol of sodium excretion in 24 h (40). Thus, temperature and the amount of salt in the diet may explain the distinct hypertension prevalence in the northern and southern regions.

Income has been identified as a hypertension risk factor (41), and our study supports these findings. We identified a positive correlation between the prevalence of hypertension and per capita GDP by province. In Bengal, adults in richer household wealth quintiles had a significantly higher prevalence and odds of hypertension (42), and women in the highest wealth quantile were more prone to hypertension in Kenya (43). In developing countries, generally, hypertension is positively correlated with economic status, but the opposite is true in many developed countries, such as the United States and Canada (44). It was revealed that higher income, occupation, and the mother’s education level were protective factors for hypertension among African Americans (45). Diet might also play a key role in influencing blood pressure by income. In developed countries, individuals with high socioeconomic status (based on occupation, education, and income) mainly consume foods abundant in fiber and protein and low in fat (46). In China, dietary consumption patterns are changing; the consumption of vegetable oil, animal foods, and sweeteners is increasing, and the consumption of coarse grains and beans is decreasing (47), especially among wealthier individuals (48). Vegetables might help reduce blood pressure (49, 50), and vegans and vegetarians have been shown to have lower SBP and DBP values than omnivores (51). A national cross-sectional study among Chinese adolescents aged 13–17 years found that adolescents whose daily vegetable consumption was three or more servings (one serving is approximately one cup, approximately 200 g) had a lower hazard ratio for high blood pressure than those who consumed less than one serving daily (52). Another study reported that both raw (tomatoes, carrots, and shallots) and cooked (tomatoes, peas, and celery) vegetable intake significantly affected blood pressure (53). Conversely, a Korean study found that vegetable intake did not influence the risk of hypertension (54), which might be owing to the manner of cooking. The Dietary Approaches to Stop Hypertension Diet, comprising whole cereal, vegetables, fruits, and low-fat food, was as effective as some antihypertensive drugs and significantly reduced blood pressure (55). Not only vegetables but also seafoods lessened the risk of hypertension. The inverse relationship was identified between high seafood intake and childhood hypertension in Iranian students aged 7–12 years (56). Seafood is abundant in omega-3 polyunsaturated fatty acids, resulting in a small but significant decrease in blood pressure (57). Moreover, one study found that people in the highest quarter of the Omega-3 Index had an SBP and DBP 4 mmHg and 2 mmHg lower, respectively, than those in the lowest quarter (58). It was noteworthy that obtaining more omega-3 polyunsaturated fatty acids from the diet led to a clinically related decrease in DBP in a randomized controlled trial (59). Since the KD-endemic areas all lie within the agricultural hinterland, increasing the intake of seafood is widely advocated for preventing hypertension in the affected population and may be an important control strategy.

This study has some limitations. First, only those aged 20 or older were included owing to the 2012 China Statistical Yearbook age group classifications; adults aged 18 and 19 years were not included. Second, the survey included many participants from several rural areas of China. Thus, we did not collect information on the participants’ hypertension drug use.

In conclusion, the prevalence of hypertension was higher in the KD-endemic areas than in the non-endemic areas. Therefore, healthy diets, such as high consumption of vegetables and seafoods, and foods that are rich in selenium, might help prevent and control hypertension in the KD-endemic areas. In addition, this study provides a better understanding of hypertension statuses in rural China, which may help with prevention.

## Data availability statement

The datasets presented in this article are not readily available because the data supporting the results of this study were obtained from the Center for Endemic Disease Control, Chinese Center for Disease Control and Prevention. The data were licensed to be used in the current study, but sharing of data was not allowed; therefore, the data resource is not publicly available. Nonetheless, the data can be available upon rational demand and with the approval of the Center for Endemic Disease Control. Requests to access the datasets should be directed to JH, houjie@ems.hrbmu.edu.cn.

## Ethics statement

The studies involving human participants were reviewed and approved by the Medical Ethics Committee of Harbin Medical University. The patients/participants provided their written informed consent to participate in this study.

## Author contributions

JH and TW performed the design and concretization of the study. JH, LZ, and SJ performed the data analysis and participated in the writing of manuscript and revision and result interpretation. JH, JSL, ZX, YW, XYW, XG, AW, XHW, JML, JM, SZ, XZ, HZ, JW, HF, and SS contributed to the field investigation and data collection. TW contributed to the project funds. All authors read the final version and approved it.

## Footnotes

1
https://www.rstudio.com/products
