# Supplementary material for: Prevalence of hypertension in endemic and non-endemic areas of Keshan disease: A cross-sectional study in rural areas of China
Source: Front Nutr. 2023 Feb 13;10:1086507. doi: 10.3389/fnut.2023.1086507 (PMC9969988; doi:10.3389/fnut.2023.1086507)
Supplement: Supplementary file 1 [file Image_1.pdf]

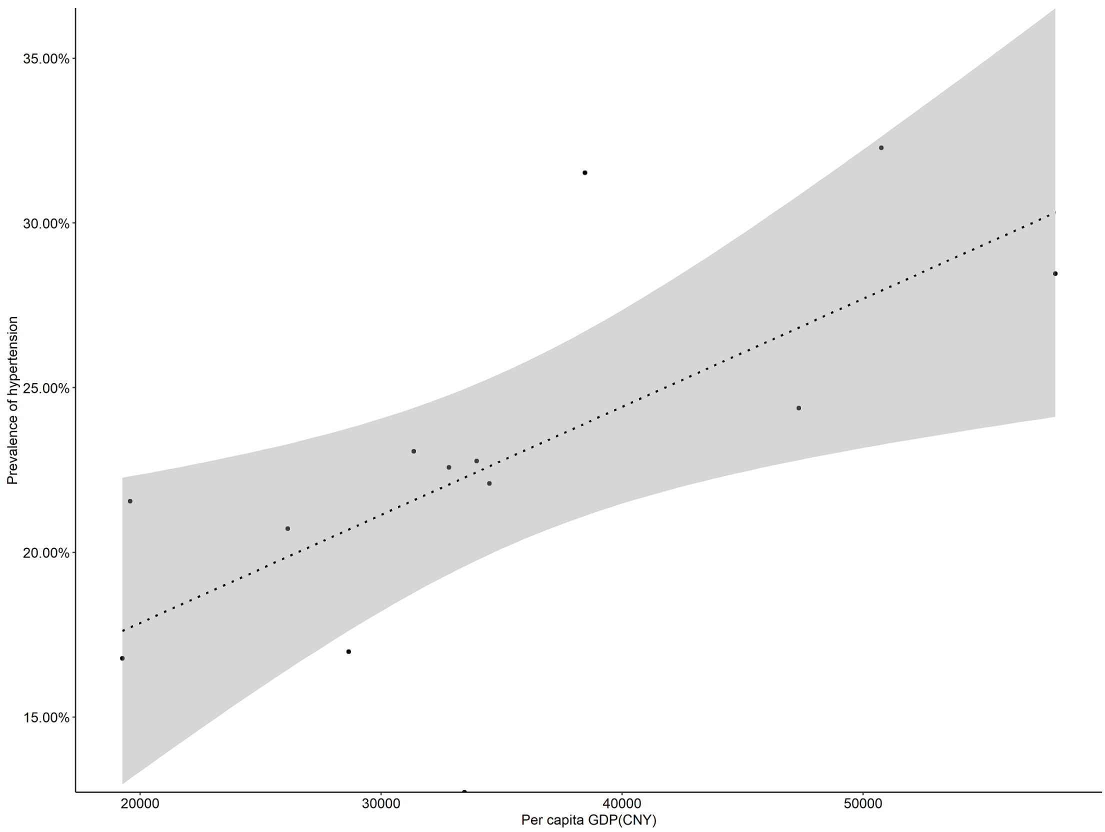

Supplemental figure 1

Hypertension prevalence and per capita GDP at province level. Scatter plots were employed to demonstrate the correlation between per capita GDP and hypertension prevalence at province level. Grey parts represent the 95% confidence interval. The prevalence was standardized by age and sex based on the 2012 China Statistical Yearbook. GDP, gross domestic product.
