# Supplementary material for: Prevalence of hypertension in endemic and non-endemic areas of Keshan disease: A cross-sectional study in rural areas of China
Source: Front Nutr. 2023 Feb 13;10:1086507. doi: 10.3389/fnut.2023.1086507 (PMC9969988; doi:10.3389/fnut.2023.1086507)
Supplement: Supplementary file 3 [file Image_3.pdf]

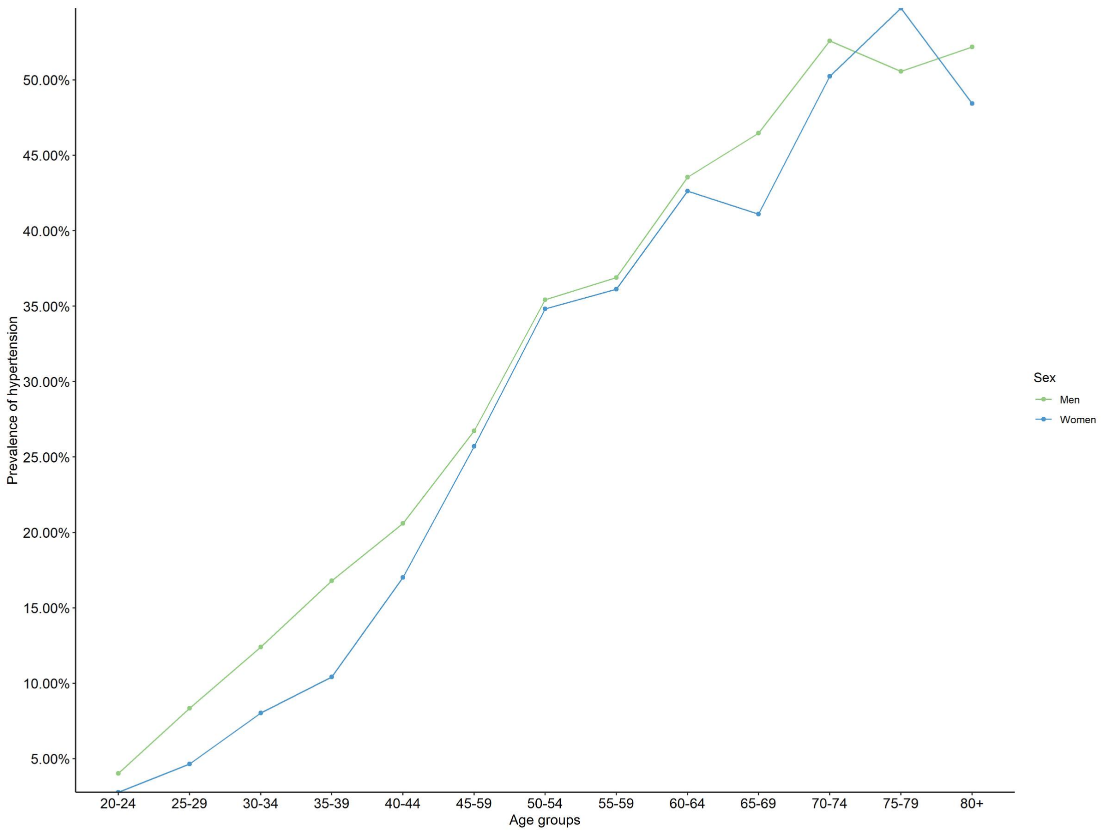

Supplemental figure 3

Hypertension prevalence in KD-endemic areas by sex and age groups. The prevalence was standardized by age and sex based on the 2012 China Statistical Yearbook. KD, Keshan disease.
