# Supplementary material for: Prevalence of hypertension in endemic and non-endemic areas of Keshan disease: A cross-sectional study in rural areas of China
Source: Front Nutr. 2023 Feb 13;10:1086507. doi: 10.3389/fnut.2023.1086507 (PMC9969988; doi:10.3389/fnut.2023.1086507)
Supplement: Supplementary file 4 [file Table_1.pdf]

Supplemental table 1. **Hypertension prevalence in KD-endemic and non-endemic areas by age groups**

Age groups	KD areas	Participants	Patients with hypertension	Prevalence (95% CI)	Age, sex-standardized prevalence (95% CI)	*P* value
20-24	Endemic	1521	51	3.35%(2.51-4.39%)	3.42%(2.56-4.46%)	<0.001
	Non-endemic	1276	86	6.74%(5.43-8.26%)	6.77%(5.43-8.26%)	
25-29	Endemic	1729	110	6.36%(5.26-7.62%)	6.51%(5.42-7.81%)	0.9561
	Non-endemic	1525	94	6.16%(5.01-7.49%)	6.42%(5.25-7.78%)	
30-34	Endemic	1991	199	9.99%(8.71-11.40%)	10.26%(8.95-11.66%)	0.5100
	Non-endemic	1780	163	9.16%(7.86-10.59%)	9.53%(8.22-11.01%)	
35-39	Endemic	2472	329	13.31%(11.99-14.71%)	13.68%(12.34-15.09%)	0.1761
	Non-endemic	2535	302	11.91%(10.68-13.23%)	12.36%(11.09-13.69%)	
40-44	Endemic	3360	622	18.51%(17.21-19.87%)	18.85%(17.53-20.20%)	0.1875
	Non-endemic	3470	596	17.18%(15.93-18.47%)	17.57%(16.33-18.89%)	
45-59	Endemic	3436	898	26.14%(24.67-27.64%)	26.23%(24.76-27.73%)	0.0780
	Non-endemic	3889	963	24.76%(23.41-26.15%)	24.39%(23.06-25.78%)	
50-54	Endemic	2600	912	35.08%(33.24-36.95%)	35.13%(33.28-36.99%)	<0.001
	Non-endemic	2815	817	29.02%(27.35-30.73%)	28.72%(27.04-30.41%)	
55-59	Endemic	3357	1224	36.46%(34.83-38.11%)	36.52%(34.89-38.18%)	0.0284
	Non-endemic	3654	1240	33.94%(32.40-35.49%)	33.99%(32.45-35.55%)	
60-64	Endemic	3012	1296	43.03%(41.25-44.82%)	43.09%(41.32-44.89%)	0.0011
	Non-endemic	3454	1349	39.06%(37.42-40.71%)	39.05%(37.42-40.71%)	
65-69	Endemic	2392	1043	43.60%(41.60-45.62%)	43.79%(41.77-45.79%)	0.6988
	Non-endemic	2466	1101	44.65%(42.67-46.63%)	44.37%(42.39-46.35%)	
70-74	Endemic	1667	856	51.35%(48.92-53.77%)	51.41%(48.98-53.83%)	0.0406
	Non-endemic	1936	931	48.09%(45.84-50.34%)	47.91%(45.69-50.19%)	
75-79	Endemic	890	469	52.70%(49.36-56.02%)	52.78%(49.47-56.13%)	0.0781
	Non-endemic	983	478	48.63%(45.46-51.80%)	48.62%(45.46-51.80%)	
80+	Endemic	311	163	52.41%(46.70-58.08%)	52.34%(46.70-58.08%)	0.9240
	Non-endemic	473	244	51.59%(46.98-56.17%)	51.72%(47.19-56.38%)	

CI, Confidence interval; KD, Keshan Disease.
