# Supplementary material for: Prevalence of hypertension in endemic and non-endemic areas of Keshan disease: A cross-sectional study in rural areas of China
Source: Front Nutr. 2023 Feb 13;10:1086507. doi: 10.3389/fnut.2023.1086507 (PMC9969988; doi:10.3389/fnut.2023.1086507)
Supplement: Supplementary file 5 [file Table_2.pdf]

Supplemental table 2. Hypertension prevalence in KD-endemic and non-endemic areas by province

Province	KD areas	Participants	Patients with hypertension	Prevalence (95% CI)	Age, sex-standardized prevalence (95% CI)	*P* value
Jilin	Endemic	2773	1124	40.53%(38.70-42.39%)	32.17%(30.43-33.94%)	0.3129
	Non-endemic	2824	1214	42.99%(41.15-44.84%)	30.87%(29.18-32.62%)	
Heilongjiang	Endemic	3547	1105	31.15%(29.63-32.71%)	25.79%(24.36-27.27%)	<0.001
	Non-endemic	3914	840	21.46%(21.80-22.78%)	19.36%(18.14-20.64%)	
Nei Mongol	Endemic	1982	765	38.60%(36.45-40.78%)	27.68%(25.74-29.73%)	0.2904
	Non-endemic	2065	718	34.77%(32.71-36.87%)	29.24%(27.29-31.26%)	
Liaoning	Endemic	606	246	40.59%(36.66-44.62%)	32.02%(28.31-35.89%)	0.8797
	Non-endemic	620	260	41.94%(38.02-45.93%)	32.53%(28.90-36.43%)	
Gansu	Endemic	3548	899	25.34%(23.91-26.80%)	21.63%(20.27-23.01%)	0.9060
	Non-endemic	3567	923	25.88%(24.45-27.35%)	21.47%(20.14-22.86%)	
Shandong	Endemic	1982	529	26.69%(24.75-28.70%)	22.59%(20.78-24.51%)	0.0072
	Non-endemic	2436	788	32.35%(30.49-34.25%)	26.16%(24.41-27.94%)	
Shanxi	Endemic	606	221	36.47%(32.63-40.44%)	28.60%(24.98-32.32%)	<0.001
	Non-endemic	932	180	19.31%(16.83-22.00%)	17.55%(15.20-20.20%)	
Shaanxi	Endemic	2696	335	12.43%(11.20-13.73%)	10.15%(9.05-11.37%)	<0.001
	Non-endemic	2839	572	20.15%(18.69-21.67%)	15.29%(13.98-16.66%)	
Henan	Endemic	765	225	29.41%(26.20-32.78%)	23.73%(20.81-26.97%)	<0.001
	Non-endemic	733	112	15.28%(12.75-18.09%)	10.23%(8.13-12.66%)	
Hebei	Endemic	1501	455	30.31%(28.00-32.71%)	23.83%(21.71-26.09%)	0.1863
	Non-endemic	1422	402	28.27%(25.94-30.69%)	21.71%(19.61-23.97%)	
Chongqing	Endemic	997	389	39.02%(35.98-42.12%)	31.11%(28.22-34.07%)	<0.001
	Non-endemic	1013	160	15.79%(13.60-18.19%)	13.07%(11.06-15.26%)	
Yunnan	Endemic	2396	582	24.29%(22.58-26.06%)	17.18%(15.70-18.77%)	0.4844
	Non-endemic	2523	614	24.34%(22.67-26.06%)	16.39%(14.98-17.91%)	
Sichuan	Endemic	5339	1297	24.29%(23.15-25.47%)	18.86%(17.82-19.94%)	<0.001
	Non-endemic	5368	1581	29.45%(28.23-30.69%)	22.58%(21.47-23.72%)	

CI, Confidence interval; KD, Keshan Disease.
