# Supplementary material for: Prevalence of hypertension in endemic and non-endemic areas of Keshan disease: A cross-sectional study in rural areas of China
Source: Front Nutr. 2023 Feb 13;10:1086507. doi: 10.3389/fnut.2023.1086507 (PMC9969988; doi:10.3389/fnut.2023.1086507)
Supplement: Supplementary file 6 [file Table_3.pdf]

Supplemental table 3. Hypertension prevalence and per capita GDP at province level

Province	Per capita GDP(CNY)	Age, sex-standardized prevalence
Jilin	38460	31.52%
Heilongjiang	32819	22.58%
Nei Mongol	57974	28.46%
Liaoning	50760	32.28%
Gansu	19595	21.55%
Shandong	47335	24.38%
Shanxi	31357	23.07%
Shaanxi	33464	12.72%
Henan	28661	16.98%
Hebei	33969	22.77%
Chongqing	34500	22.09%
Yunnan	19265	16.78%
Sichuan	26133	20.72%
